# Cell spheroids are as effective as single cells suspensions in the treatment of critical-sized bone defects

**DOI:** 10.1186/s12891-021-04264-y

**Published:** 2021-04-30

**Authors:** Lisa Findeisen, Julia Bolte, Corina Vater, Cathleen Petzold, Mandy Quade, Lars Müller, Stuart B. Goodman, Stefan Zwingenberger

**Affiliations:** 1 University Center for Orthopedic, Trauma and Plastic Surgery, University Hospital Carl Gustav Carus, TU Dresden, Dresden, Germany; 2 Center for Translational Bone, Joint and Soft Tissue Research, University Medicine Carl Gustav Carus Dresden, TU Dresden, Dresden, Germany; 3 Department of Orthopedic Surgery, Stanford University, Stanford, USA

**Keywords:** Critical-sized bone defect, Bone regeneration, Mesenchymal stromal cells, Cell spheroids

## Abstract

**Background:**

Due to their multilineage potential and high proliferation rate, mesenchymal stem cells (MSC) indicate a sufficient alternative in regenerative medicine. In comparison to the commonly used 2-dimensional culturing method, culturing cells as spheroids stimulates the cell-cell communication and mimics the in vivo milieu more accurately, resulting in an enhanced regenerative potential. To investigate the osteoregenerative potential of MSC spheroids in comparison to MSC suspensions, cell-loaded fibrin gels were implanted into murine critical-sized femoral bone defects.

**Methods:**

After harvesting MSCs from 4 healthy human donors and preculturing and immobilizing them in fibrin gel, cells were implanted into 2 mm murine femoral defects and stabilized with an external fixator. Therefore, 26 14- to 15-week-old nu/nu NOD/SCID nude mice were randomized into 2 groups (MSC spheroids, MSC suspensions) and observed for 6 weeks. Subsequently, micro-computed tomography scans were performed to analyze regenerated bone volume and bone mineral density. Additionally, histological analysis, evaluating the number of osteoblasts, osteoclasts and vessels at the defect side, were performed.

Statistical analyzation was performed by using the Student’s t-test and, the Mann-Whitney test. The level of significance was set at *p* = 0.05.

**Results:**

μCT-analysis revealed a significantly higher bone mineral density of the MSC spheroid group compared to the MSC suspension group. However, regenerated bone volume of the defect side was comparable between both groups. Furthermore, no significant differences in histological analysis between both groups could be shown.

**Conclusion:**

Our in vivo results reveal that the osteo-regenerative potential of MSC spheroids is similar to MSC suspensions.

**Supplementary Information:**

The online version contains supplementary material available at 10.1186/s12891-021-04264-y.

## Article summary

Article focus

- This study investigated the osteo-regenerative potential of MSC spheroids in comparison to MSC suspensions in a critical-sized defect.

## Key messages

- The regenerated bone mineral density of the MSC spheroid group is higher than the MSC suspension group.
- The regenerated bone volume of the MSC spheroid group was similar to the MSC suspension group.
- Histological degree of defect healing, as well as bone cell markers, showed no significant difference between the 2 groups.
- Fibrin gel-embedded MSCs, independent of their condition in the gel, are not able to heal a critical-sized defect.

## Strengths and limitations of this study

- MSCs from 4 individual donors, which were gender-balanced (2 male, 2 female), were used. No donor-dependent effect could be observed as shown in supplementary data.
- A well-established surgical method was used, providing standardized results.
- By using the fibrin gel as an implant, a standardized carrier for cells was provided.
- The number of cells per spheroid might have led to tightened packaging and less available nutrition in the spheroid core.
- No biomechanical testing was performed of dissected femora since defects were not bridged.

## Introduction

Critical-sized bone defects can result from wound infections with extensive debridement, complicated fractures with high bone loss, or tumour resections which are challenging to treat. Implantation of autologous bone, generally harvested from the patient’s iliac crest, is still the gold standard because of its favourable osteoconductive, osteoinductive and cellular properties [1]. However, autologous graft is accompanied by disadvantages including potential tissue morbidity and limited bioavailability [2, 3]. Alternatively, bone replacement materials or cell-based therapies might be used due to their promising therapeutic outcomes. Mesenchymal stromal cells (MSCs) are multipotent cells that are located in all organs having connective tissue [4], including adult bone marrow, umbilical cord blood, fetal liver and fat [5–7]. Their osteogenic potential is based on the high proliferation rate, low immunogenicity and their multilineage capacity [4, 8, 9]. Therefore, functional cells like osteoblasts and chondrocytes can be released at the defect side. Defect healing is improved by the secretome of MSCs, consisting of growth factors, cytokines and angiogenic factors [10]. Cryopreserved cells can certainly be used, since preservation and thawing do not influence growth and osteogenic differentiation [11]. However, disadvantages of MSCs have been revealed, such as the difficulty of maintaining cell functions, the attachment to host cells, as well as their low survival rate during transplantation [12–14]. The unphysiological microenvironment, originating from culturing cells with the commonly used 2-dimensional culture technique, might be an explanation for these disadvantages [15]. Attempts have been made to imitate the complex, physiological milieu of cells and tissues, leading to the development of 3-dimensional culture systems. In this regard, increased overall functions could be observed when forming multicellular aggregates, as cell-cell interactions are improved [16–18]. MSC spheroids show enhanced osteoinductive properties in vivo and in vitro due to their interaction with an endogenous environment and extracellular matrix and their retainment of osteogenic differentiation [16, 19–21]. Furthermore, the unique culture geometry also induces anti-inflammatory, anti-apoptotic and pro-angiogenic effects [17, 18, 22–24]. Osteogenesis and angiogenesis promoting properties are required in particular by pathological bone healing, for example when avascularity and hypoxia occur in large bone defects [25]. All in all, these improvements might be beneficial to the treatment of critical-sized defects with their relatively harsh microenvironment. There are several definitions for a critical-sized bone defect. In general, for segmental bones the length of a critical-sized defect is about 1.5 times the diameter of the respective bone [26]. When establishing the murine femoral critical-sized bone defect model, which is used in this study, we could show that in the 2 mm defect group only 3 out of 8 bones healed [27]. Therefore, we have chosen the 2 mm defect. Furthermore, when the defect was filled with a cell-free mineralized collagen scaffold, we could not observe sufficient bone growth in previous studies [28].

We hypothesized that MSC spheroids would show a higher bone regenerative potential in a critical-sized femoral bone defect model in mice compared to MSC suspensions.

## Material and methods

### Study design

**Fig. 1:**
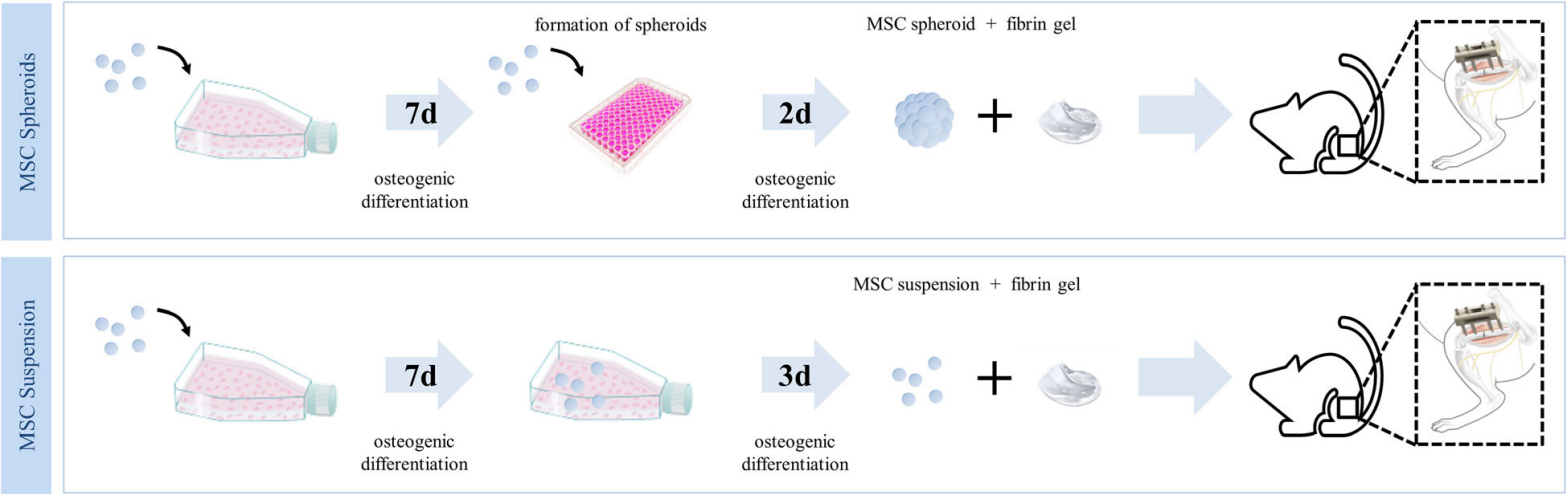
Study design. MSCs were isolated from human bone marrow aspirates from healthy donors, divided into 2 groups: (1) MSC spheroids and (2) MSC suspensions and osteogenically pre-differentiated for 7 days. For spheroid formation, the detached MSC suspension was resuspended in osteogenic medium and transferred into 96-well suspension plates, followed by an incubation of 2 days. MSC suspensions were osteogenically differentiated in culture flasks for another 3 days. Then, 5‧10^4^ cells - either as suspension or as spheroid - were immobilized within fibrin gels and implanted into a 2 mm segmental bone defect in the right femora of 26 nu/nu NOD/SCID nude mice that was stabilized by an external fixator. Animals were observed for 6 weeks. Afterwards, μCT and histological analyses were performed

Firstly, to immobilize the cells within the defect they were encapsulated in fibrin gels. Therefore, bone marrow aspirates were harvested from healthy human donors undergoing total hip arthroplasty. After isolation of the MSCs, cryopreservation and thawing, MSCs were cultured and pre-differentiated in osteogenic medium. Cells were divided in 2 groups (MSC spheroids, MSC suspensions) and generated implants underwent a qualitative live-dead staining test. To verify the survival rate of cells in vivo is a major challenge. To show that spheroidal and suspension cells immobilized within the fibrin gel were alive at the time of implantation, we performed live/dead staining. MSC spheroids were encapsulated into the fibrin gels after 9 days of culture; MSC suspensions were encapsulated after 10 days (Fig. 1).

Secondly, 26 nu/nu NOD/SCID nude mice were randomized into 2 groups (MSC spheroids, MSC suspensions). A critical-sized defect of 2 mm was created on the right femur of all animals and stabilized with an external fixator. The fibrin gel was implanted into the defect according to group allotment. After an observation period of 6 weeks, animals were euthanized and high-resolution micro-computed tomography (μCT) as well as histological analysis were performed on all explanted right femora.

### Preparation of MSCs

MSCs were isolated after informed consent (ethics approval number: EK91032012) from human bone marrow aspirates of 4 healthy donors (2 male: 70 and 71 years old, 2 female: 71 and 79 years old) undergoing total hip arthroplasty by density gradient centrifugation (Ficoll-Paque Plus, GE Healthcare, 1.077 g/ml, Little Chalfont, United Kingdom) and subsequent plastic adherence. Due to logistic reasons and to make culture conditions comparable, cyropreserved cells were used for implant preparation. Therefore, MSCs of passage 2 were thawed and cultured in basic medium (DMEM, 15% FCS, 1% penicillin/streptomycin) for 3 to 4 days until they reached confluency. Then, basic medium was changed to osteogenic medium (DMEM, 10% FCS, 1% penicillin/streptomycin, 100 nM dexamethasone, 10 mM beta-glycerolphosphate, 50 mM ascorbic acid-2 phosphate) for osteogenic pre-differentiation for 7 days. Exchange of medium was performed twice a week.

### Mesenchymal stem cell spheroid formation

The pre-differentiated cells were detached from the culture flasks using 0.5% trypsin/ ethylenediaminetetraacetic acid (EDTA), counted and resuspended in osteogenic medium to obtain a single cell suspension with a concentration of 0.25‧10^6^/ml. 200 μl of this cell suspension (= 5‧10^4^ cells) were added per well to a 96-well suspension plate (Sigma-Aldrich, St. Louis, USA) and centrifuged for 5 min at 200 g at room temperature to form one spheroid. Cells were then incubated at 37 °C and 5% CO_2_ for 2 days.

### Preparation of the implants

Cylindrical implants (Ø: 2 mm, length: 2 mm) were produced using fibrin gel (TISSEEL, Baxter, Frankfurt, Germany) containing 45 mg/ml of fibrinogen and 250 IU/ml of thrombin. To prepare spheroid-loaded implants, 15 μl of the fibrinogen solution were pipetted into 0.5 ml tubes. Spheroids in the 96-well plate were washed once with PBS and then 1 spheroid (containing 5‧10^4^ cells) was carefully transferred using a pipette tip into the 0.5 ml tube. After adding 15 μl of the thrombin solution, fibrin gels were allowed to polymerize for 30 min at 37 °C. Gels were carefully stirred using a 10 μl pipette tip and incubated for another 30 min.

To prepare suspension-loaded implants, pre-differentiated cells were detached from the culture flasks using 0.5% trypsin/EDTA, counted and resuspended in fibrinogen to a concentration of 3.33‧10^6^/ml. 15 μl of this cell suspension were mixed with 15 μL thrombin solution and allowed to polymerize as mentioned above.

After polymerization, solid fibrin gels were transferred into 2 ml tubes and immediately used for implantation. The number of spheroid- and suspension-loaded implants prepared from MSCs of the 4 individual donors that were implanted into the animals is shown in Table 1.

**Table 1:** Number of implants prepared from the 4 individual MSC donors

MSC implants	donor 1	donor 2	donor 3	donor 4
spheroid-loaded implants	3	3	3	3
suspension-loaded implants	3	4	3	4

### Cell viability within implants

Two additional implants per donor and group were prepared in the above-mentioned way and used - right after producing the implants - to check viability of cells before implantation by live/dead and MTT staining. For MTT staining that detects metabolic activity of cells, the implants were incubated with 0.5 mg/ml 3-(4,5-dimethylthiazol-2-yl)-2,5-diphenyltetrazolium bromide (MTT, Sigma Aldrich, St. Louis, USA) in basic medium for up to 4 h at 37 °C. After washing with PBS, implants were fixed with 4% neutral buffered formaldehyde and imaged using a stereo microscope Leica M125 C (Leica Microsystems AG, Heerbrugg, Switzerland).

Live and dead cells within the implants were visualized using the Live/Dead Viability/Cytotoxicity Kit (ThermoFisher Scientific, Waltham, USA). Therefore, implants were washed once with PBS and subsequently incubated with 2.4 μM calcein AM (live cells) and 2.4 μM ethidium homodimer-1 (dead cells) in PBS. After light-protected incubation for 30 min at 37 °C, implants were washed once with PBS and cell viability was examined immediately using a Keyence BIOREVO BZ-9000 microscope (Keyence, Neu-Isenburg, Germany) with ex/em: 495/515 nm for calcein and 495/635 nm for ethidium homodimer-1.

### Animals

For the in vivo study, 26 male, 14–15 weeks old nu/nu NOD/SCID nude mice (35.8 ± 3.2 g) were randomized into 2 groups. The mice were bred at the Centre for Regenerative Therapies (CRTD), Technische Universität Dresden, fed with standard diet (food and water ad libitum) and kept at a 12-h light and dark cycle. All animal experiments were performed in accordance to the National Institutes of Health Guidelines for the Use of Experimental Animals and were approved by the Local Animal Care and Ethics Committee of Dresden University Hospital (protocol no. 24–9168.11-1/2013–75). All of the 26 animals survived the surgeries and the observation period.

### Surgical procedure

Surgery was performed under 2% isoflurane anesthesia as described previously [27]. Additionally, each animal received 300 μl saline with 1.6 μg buprenorphine as s. c. injection to minimalize pain. After placing the animal in prone position, a 12 mm incision was made along the lateral tight. To expose the femur, the incision was extended through the *fascia lata*, reaching from the great trochanter to the knee joint. Subsequently to the femur exposure and mobilization of muscles, an external fixator (MouseExFix simple XL, RISystem, Landquart, Switzerland) was placed by drilling pins into the lateral and medial cortex. The 2 mm defect was created with a 2 mm saw guide (RISystem) and Gigli wires (0.22 mm, RISystem). Thereafter, depending on the group allotment, implants were placed into the defect. Femur and surrounding tissue were relocated to their physiological position and the skin was closed by Donati suturing technique (Ethilon 4–0, Ethicon, Johnson & Johnson, New Brunswick, NJ).

### Preparations for high-resolution micro-computed tomography and histology

After 6 weeks of observation animals were sacrificed by exposure to CO_2_ following cervical dislocation. Right and left femora were dissected, cleaned from soft tissue and placed into 15 ml tubes filled with 4% neutral buffered formaldehyde that was changed every 2 days. μCT scanning of all femora was performed under these conditions. To decalcify the bones, formaldehyde was replaced with EDTA. After dehydration using an ascending ethanol series, paraffin was used to embed the femora and samples were sagittally cut into slices of 2 μm.

### High-resolution micro-computed tomography - μCT analysis

All femora were scanned with a SCANCO vivaCT 40 (Scanco Medical AG, Wangen-Brüttisellen, Switzerland). According to the manufacturer’s instructions, the following calibration steps were performed: 5 rods of different hydroxyapatite densities were scanned once per week. To additionally check the alignment of the device, 3 aluminium wires were scanned once per month.

The following μCT settings were used to scan the mice femora: X-ray intensity = 145 μA, X-ray tube = 55 kVp, voxel size = 21 μm, integration time = 200 ms, projections = 1000.

Analysis was performed by using the software of the SCANCO vivaCT 40. Therefore, a standardized 3D-region of interest with cylindrical shape (Ø: 2.5 mm, length: 3.5 mm) was determined with setting the centre between the inner 2 pins of the external fixator. Bone tissue density was defined as > 200 mg hydroxyapatite/cm^3^ and bone volume was measured in mm^3^.

### Histological examination

All histological sections were evaluated using a Keyence BIOREVO BZ-9000 microscope (Keyence, Neu-Isenburg, Germany).

Firstly, the grade of defect healing according to Huo et al. was classified by 3 different, blinded observers [29]. Therefore, tissue sections were stained with haematoxylin and eosin (H&E, Merck, Darmstadt, Germany) and 3 representative sections per femur were assessed.

Secondly, vascularization and bone cell markers of the defect area were analysed. The area of interest was standardised by detecting the area between the inner pin holes with a 2x objective and separating it in 12 squares with the software of the microscope (BZ-II Viewer, Keyence, Neu-Isenburg, Germany).

To evaluate vascularization, alpha-smooth muscle actin staining (rabbit anti-smooth muscle actin, 1:750, Cat.# M0851, Agilent Dako, Santa Clara, USA) was used. Vessels in the defect area showing a lumen were counted, vessels in the surrounding muscle and soft tissue were omitted.

To evaluate bone cell markers, slides were stained with bone alkaline phosphatase (BAP, anti-rabbit IgG peroxidase, 1:100, Cat.# PAK0142, LINARIS Biologische Produkte GmbH, Dossenheim, Germany) for osteoblasts and tartrate resistant acid phosphatase (TSP, Sigma-Aldrich, St. Louis, USA) for osteoclasts. Number of osteoclasts and osteoblasts were counted.

### Statistics

Statistical analysis was done using GraphPad Prism 5.00 software (San Diego, CA, USA). All data are presented as mean ± standard deviation. For bone volume, bone mineral density, number of osteoblasts and osteoclasts and vascularization differences between the 2 groups were tested using the 2-sided unpaired Student’s test (normally distributed data) whereas its non-parametric equivalent Mann-Whitney test was applied for the histological degree of defect healing. The level of significance was set at *p* = 0.05.

## Results

### Cell viability within implants prior implantation

**Fig. 2:**
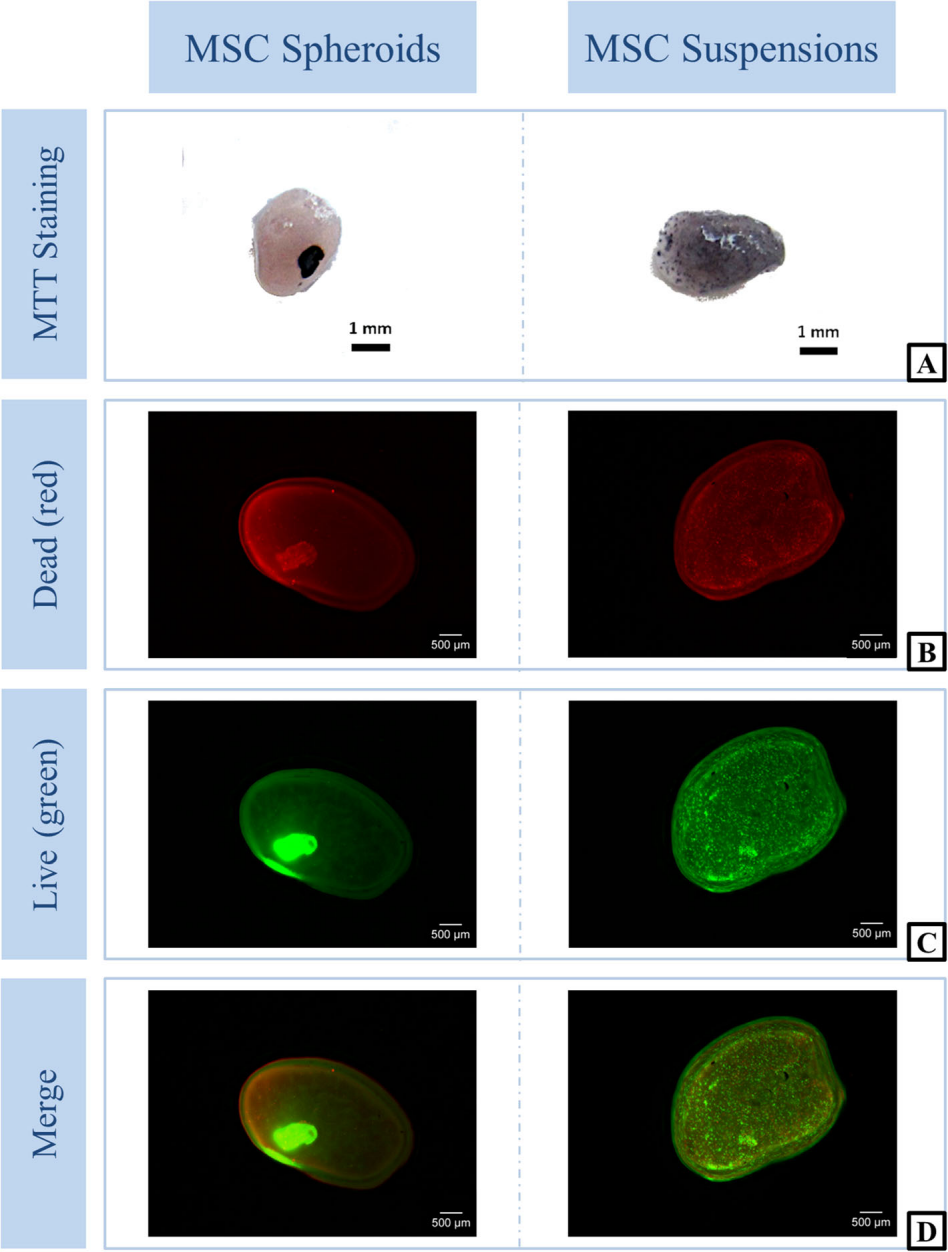
Cell viability within implants prior implantation. Representative pictures of MTT- (**a**) and live-dead-stained (**b**-**d**) implants of 1 MSC donor showing the distribution of cells in the fibrin gel implants. (MTT: dark spots = metabolically active/living cells; live-dead staining: green = living cells, red = dead cells)

As observed by MTT and live-dead staining spheroid as well as suspended cells showed good viability within the fibrin gel implants (Fig. 2).

### μCT analysis

**Fig. 3:**
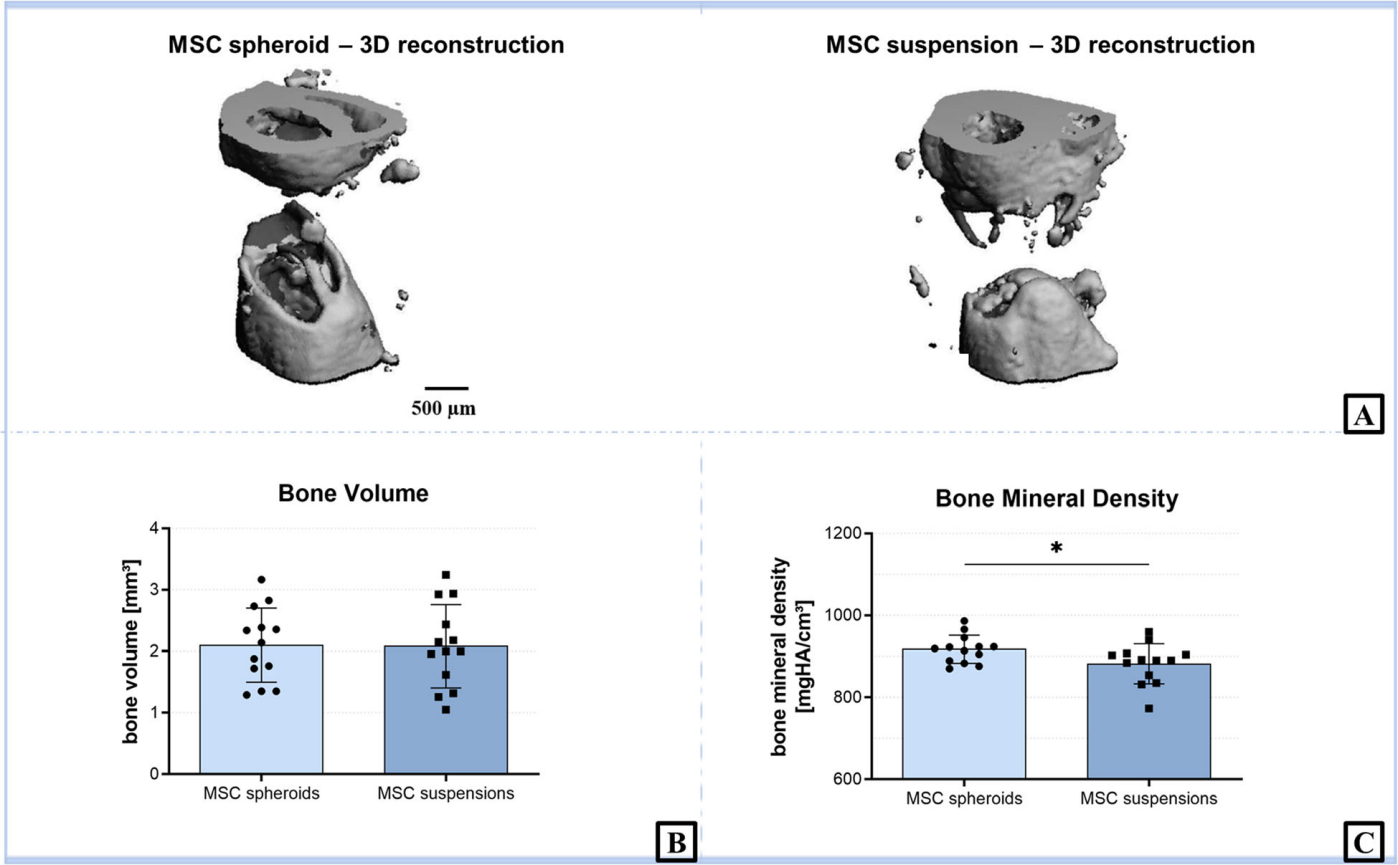
μCT-based evaluation of the defect area 6 weeks postoperatively. Representative 3D reconstruction of the defect area by high resolution μCT for MSC spheroids and suspensions (**a**) group. Bone volume (**b**) and bone mineral density (**c**) in the defect area as determined by μCT. Whereas no intergroup difference could be observed for bone volume, MSC spheroids group showed a significantly higher bone mineral density than the MSC suspensions group (mean ± SD; 2-sided Student’s t-test, * p < 0.05)

After a 6 weeks observation period, μCT analysis was performed to investigate the volume of the newly regenerated bone as well as bone mineral density (Fig. 3). Bone volume showed no intergroup difference in and around the defect area (MSC spheroids vs. MSC suspensions; 2.1 ± 0.2 mm^3^ vs. 2.1 ± 0.2 mm^3^, *p* = 0.9443). However, treating the defect with spheroid-loaded implants led to a significantly increased bone mineral density at the defect side (917 ± 9 mgHA/cm^3^) as compared to the suspension-loaded implants (882 ± 14 mgHA/cm^3^, *p* = 0.043). A systematic bias could be excluded since the difference between the bone mineral density of the un-operated femora of both groups was not significant (Additional File 1).

### Histological analysis

**Fig. 4:**
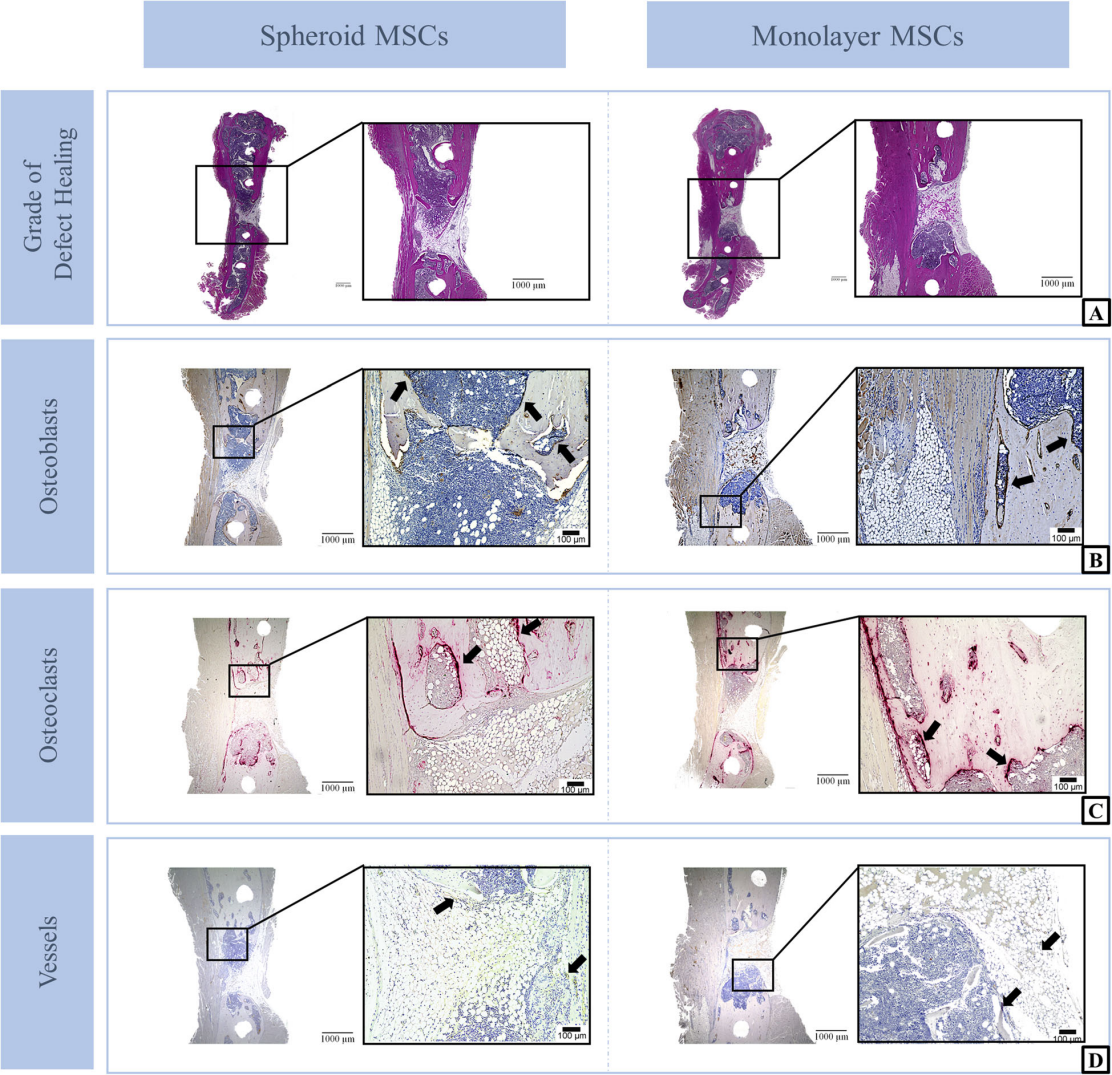
Histological staining: Representative histologically stained sections of the defect area of MSC spheroids and suspensions groups, 6 weeks after implantation. Histological grade of defect-healing is demonstrated through H&E staining (**a**). In order to investigate bone cell markers, BAP staining was performed to detect osteoblasts (**b**) and TSP staining for osteoclasts (**c**). Actin-staining was done to analyze vascularization (vessels marked by black arrows) (**d**)

Representative slides for each group and staining are shown in Fig. 4.

### Histological degree of defect healing

**Fig. 5:**
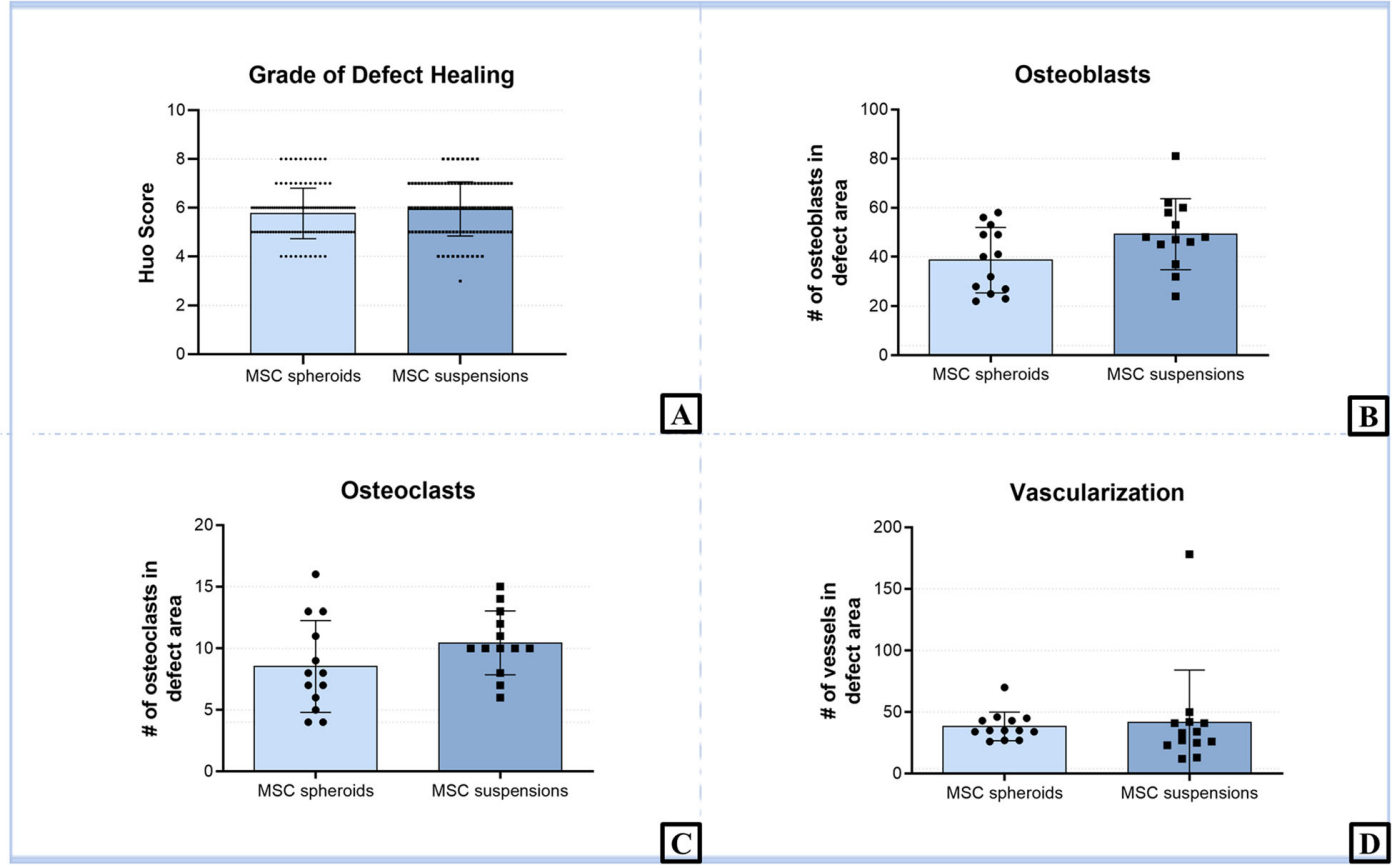
Histological analysis of bone cell markers and vascularization: To grade the defect healing (**a**), 3 stained sections per animal were observed by 3 blinded observers and rated according to Huo et al. [29] (mean ± SD, Mann-Whitney test). To investigate bone cell markers and vascularization, number of osteoblasts (**b**), osteoclasts (**c**), and vessels (**d**) were counted in the defect area 6 weeks after implantation (mean ± SD, 2-sided Student’s t-test). No significant difference could be shown between the groups

Due to the numerical scoring schema of Huo et al. for histological analysis of fracture healing, a refined evaluation of the actual bone tissue healing can be achieved. Constant with the bone volume results of the μCT analysis, no difference in the degree of defect healing between the groups (MSC spheroids vs. MSC suspensions; 5.77 ± 1.04 vs. 5.95 ± 1.12; *p* = 0.1231) could be shown (Fig. 5a). Additionally, no bridging of the defect area of any dissected femora could be seen.

### Histological analysis of bone cell markers and vascularization

To investigate bone cell markers, the number of osteoblasts and osteoclasts were counted in the defect area (Fig. 5b - d). Neither the number of osteoblasts (MSC spheroids vs. MSC suspensions; 38.69 ± 13.24 vs. 49.31 ± 14.43 cells/field; *p* = 0.0721), nor the number of osteoclasts (MSC spheroids vs. MSC suspensions; 8.54 ± 3.73 vs. 10.46 ± 2.60 cells/field; *p* = 0.2113) was significantly influenced by the condition of the cells within the implants. Furthermore, the vascularization seemed to be independent from the kind of MSC condition (MSC spheroids vs. MSC suspensions; 38.46 ± 11.65 vs. 41.92 ± 42.41 vessels/field; *p* = 0.7842).

## Discussion

We hypothesized that MSC spheroids have a higher bone regenerative potential than MSC suspensions when applied in a critical-sized, murine femoral defect. Therefore, 5‧10^4^ osteogenically pre-differentiated human MSCs of 4 individual donors were immobilized within fibrin gels, either as 1 cell spheroid or as cell suspension, and implanted into 2 mm critical-sized femoral defects in mice. These immune deficient mice were especially chosen due to the implantation of human MSCs and to be in line with the study on which the critical-sized bone defect model was established. Previous studies also showed a regular bone growth and bone healing [27, 30].

Micro-CT analysis revealed a significantly higher bone density of the MSC spheroids group compared to the MSC suspensions group. MSC donor status did not correlate with bone mineral density and bone volume (Additional File 2). However, the regenerated bone volume at the defect side treated with MSC spheroids was comparable to the one treated with MSC suspensions. Additionally, histological analysis showed no significant differences regarding bone cell markers (number of osteoblasts and osteoclasts), as well as vascularization at the defect side for both groups.

Mesenchymal stromal cells can enhance the healing of bone defects due to their multilineage potential [31, 32]. In addition to that, MSC transplantation is beneficial to critical-sized bone defect healing because MSC attract other cells like osteoblasts and chondrocytes. Especially when exposed to hypoxic conditions, MSCs start to secrete different cytokines [16]. MSCs are able to differentiate into multiple cell types such as fibroblasts, chondrocytes, adipocytes, and osteoblasts. To achieve a specific differentiation, cells must be treated with special media e.g. osteogenic medium to guide them to the osteogenic lineage [33, 34]. It was shown that both, spheroid and suspension cells, differentiate into osteoprogenitor cells [16, 23]. The success of culturing MSCs adequately depends on several factors including seeding density and pressure, thus, the same number of cells and seeding conditions were used in each group [35].

Furthermore, our previous studies on MSCs isolated from bone marrow, using the same procedure as the current study, could confirm the expression of mesenchymal stem cell markers and their trilineage differentiation potential [36, 37]. When MSCs are immobilized within spheroids, several in vitro tests, which were performed by Yamaguchi et al., showed an upregulation of the osteogenic marker genes RUNX-2, OSX, BSP and OPN as compared to monolayer cultured cells [16, 38]. Furthermore, as mentioned by Cesarz et al., MSC spheroids show an enhanced multidifferentiation potential and upregulation of pluripotency marker genes indicating enhanced stemness.

Previously, 2D culture has been commonly used to obtain osteoblasts for bone healing in multiple studies. However, these culturing techniques do not imitate the physiological microenvironment in vivo unlike culturing cells as spheroids [20, 21, 39]. Simulating the native morphology with a spheroid morphology, greater cell-cell contacts are formed leading to anti-inflammatory und anti-apoptotic effects [18, 22].

As described by Yagamuchi et al., 5‧10^4^ cells were used and spheroids were formed successfully and immobilized within fibrin gels [16]. Good viability of fibrin gel-encapsulated cells was demonstrated with MTT and live-dead staining, prior to implantation.

Fibrin gel was chosen to immobilize the cells in the bone defect due to its osteopromotive properties which influence the osteogenic potential of MSC suspension, as well as of MSC spheroids [17]. Tisseel is a clinically approved tissue sealant, can be applied minimal invasively, polymerizes fast, offers shape variability, compressive stiffness, degrades in vivo due to its similarity to physiological blood clots within around 7 days, provides a comfortable environment for the cells and allows them to immigrate [40–42]. Yet, fibrin gel does not replicate the mineralized compound and nanostructure of bone. In order to enhance mineral content, hydroxyapatite can be incorporated into fibrin gel. These scaffolds showed improved defect healing [43, 44]. However, the adhesivity should be improved, since limited cell migration of MSC spheroids leads to increased bone formation [44]. Alternatively, mineralized collagen matrix scaffolds (MCM) are a suitable substrate for MSCs [28]. By comparing our findings, in particular those referring to bone volume (MSC spheroids: 2.1 ± 0.2 mm^3^; MSC suspensions: 2.1 ± 0.2 mm^3^), to Bolte et al. (MCM only: 3.9 ± 2.0 mm^3^; MCM + pre-differentiated MSCs: 6.3 ± 1.3 mm^3^)*,* one concludes that not only pre-differentiation of MSCs, but also the type of scaffold has a significant impact on bone healing [28].

The defect into which the MSCs were implanted can be described as critical-sized since 37.5% of animals with a defect size of 2 mm showed no bridging as observed in a study performed by Zwingenberger et al. [27]. In line with Bolte et al. and Quade et al. we also used a 2 mm defect for testing our hypothesis [28, 45]. After 6 weeks of observation, the bone density of the spheroid group was increased, which might be explained by upregulated levels of expression of osteogenic genes in MSC spheroids, as observed in vitro by Yagamuchi et al. Besides, spheroids have an enhanced survival rate under ischemic conditions compared to suspended cells [24].

However, an increased healing of the defect by MSC spheroids was not shown as opposed to the application of osteoinductive materials like BMP-2 or using a 2-step stem cell therapy [28, 45, 46].

Limitations of this study are the usage of one spheroid containing 5‧10^4^ cells per implant due to technical reasons, leading to deficiencies of inter-spheroidal cell communication. Spheroids consisting of more than 15,000 cells result in diameters larger than 200 μm [17, 47]. Thus, limitations of diffusion and nutrient transport might be exceeded, creating hypoxia in the core of the spheroid [47]. Caspase activity is thereby upregulated, indicating a higher level of apoptosis [17]. Despite the high cell viability within the spheroid immediately prior to implantation, as observed by MTT and live-dead staining, this effect of hypoxia might increase over time when cells are implanted. On the other hand, there is evidence that a hypoxic core might enhance cell survival and secretion of trophic factors [48, 49]. Additionally, it has been shown that larger spheroids secret more prostaglandin E2 and vascular endothelial growth factor than smaller spheroids, which can stimulate defect healing advantageously [21].

For further investigations regarding the osteoregenerative potential of MSC spheroids, smaller spheroids containing less cells might be used. Alternatively, the gravity-driven hanging drop method could be applied as a spheroid formation technique due to its ease of use, lack of specialized equipment and utility for small spheroids [44, 50]. As a next step, genetically modified MSC spheroids showing an enhanced upregulation of migration-related genes and maintaining these qualities through pathological conditions could be implanted into critical-sized defects [51]. One could also pre-culture MSCs under hypoxic conditions that enhance therapeutic effects of spheroids [51]. Further improvements in the culture methods of MSC spheroids cultivation might prove useful to bone regeneration.

## Conclusion

With regards to the regenerated bone volume, it was shown that MSC spheroids are comparable to MSC suspensions for the treatment of a critical-sized bone defect. In contrast, using MSC spheroids led to an increased bone mineral density which could be beneficial for older patients with osteoporosis or deficient bone healing capacity. However, the osteoinductive potency of the investigated cells alone - independent from their appearance within the implant – is insufficient for healing large bone defects in contrast to established clinical methods such as autograft bone. Future improvements of MSC spheroids might lead to greater bone regenerative potential such that the limited therapeutic options of critical-sized bone defects could be successfully replaced.

## Supplementary Information

**Additional file 1.** Difference between left and right femora.

**Additional file 2.** MSC donor’s effect.

## Data Availability

The datasets used and analyzed during the current study are available from the corresponding author on reasonable request.
