# Supplementary material for: Cell spheroids are as effective as single cells suspensions in the treatment of critical-sized bone defects
Source: BMC Musculoskelet Disord. 2021 Apr 30;22:401. doi: 10.1186/s12891-021-04264-y (PMC8091496; doi:10.1186/s12891-021-04264-y)
Supplement: Supplementary file 1 — Additional file 1. Difference between left and right femora. [file 12891_2021_4264_MOESM1_ESM.docx]

**Additional File 1: Difference between left and right femora**


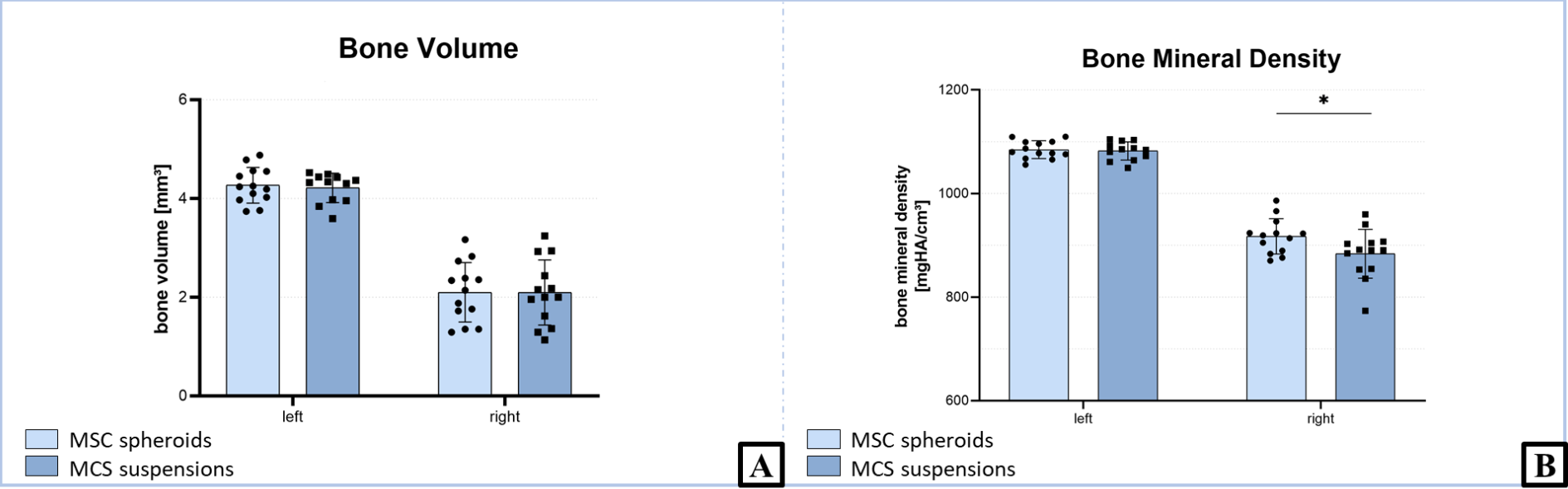


To exclude systematic bias, the bone volume (A) and bone mineral density (B) of the untreated femora of each group were compared by a 2-sided Student´s t-test, level of significance was set at p = 0.05 (mean ± SD). No significant differences between bone volume (MSC spheroids left vs. MSC suspensions left; 4.268 ± 0.3607 mm^3^ vs. 4.196 ± 0.2920; p = 0.5832) and bone mineral density (MSC spheroids left vs. MSC suspensions left; 1085 ± 17 mgHA/cm³ vs. 1082 ± 17 mgHA/cm³; p = 0.6763) could be shown.
