# Supplementary material for: Cell spheroids are as effective as single cells suspensions in the treatment of critical-sized bone defects
Source: BMC Musculoskelet Disord. 2021 Apr 30;22:401. doi: 10.1186/s12891-021-04264-y (PMC8091496; doi:10.1186/s12891-021-04264-y)
Supplement: Supplementary file 2 — Additional file 2. MSC donor’s effect. [file 12891_2021_4264_MOESM2_ESM.docx]

**Additional File 2: MSC donor´s effect**


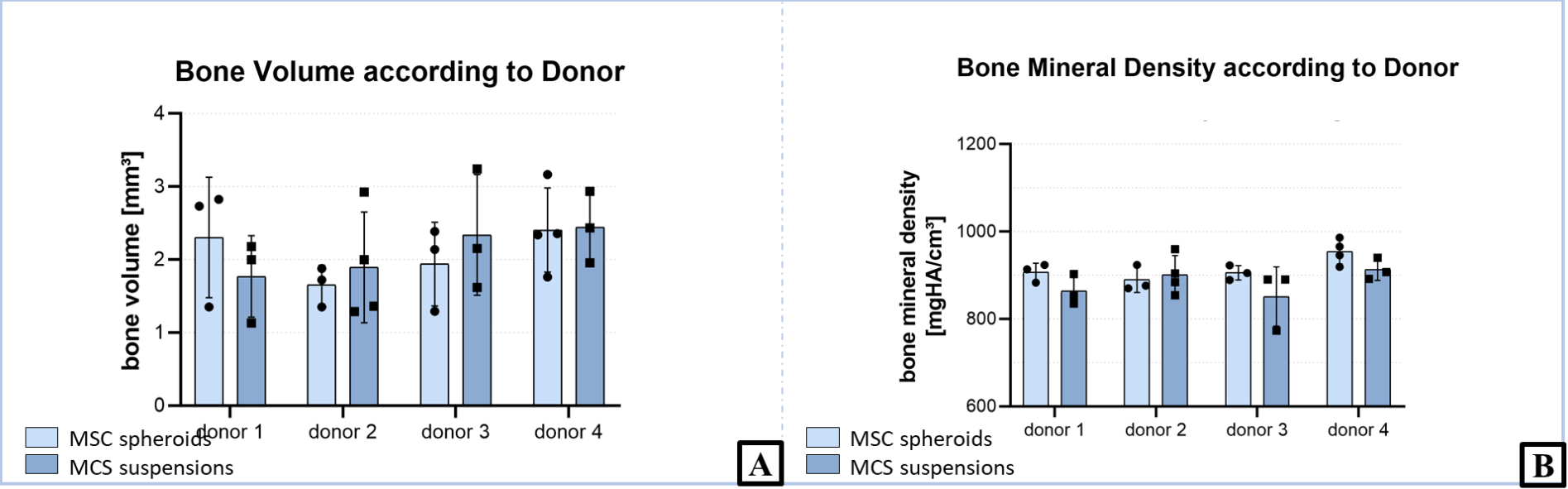


To analyze the effect of MSC donors on bone volume (A) and bone mineral density (B) compared with MSC condition, a 2-way ANOVA was conducted, level of significance was set at p = 0.05 (mean ± SD). Simple main effects analysis showed that the bone volume was not influenced by MSC condition (p = 0.8869) or MSC donor (p = 0.3339).

Also, there was no significant interaction between MSC condition and donors on bone mineral density
(p = 0.4011). Simple main effect analysis showed that MSC condition significantly increased bone mineral density (p = 0.0411), but there were no differences between MSC donors (p = 0.0609).
